# UAV path planning based on third-party risk modeling

**DOI:** 10.1038/s41598-023-49396-4

**Published:** 2023-12-14

**Authors:** Haoyang Tang, Qiang Zhu, Bo Qin, Ruoyang Song, Zhe Li

**Affiliations:** School of Automation, Xi’an University of Posts and Telecommunications, Xi’an, 710121 China

**Keywords:** Electrical and electronic engineering, Information technology, Software

## Abstract

Drones play an important role in modern cities, they bring convenience but also bring corresponding risks. Falling drones can cause casualties or damage to urban facilities and serious property damage, which increases the third-party risk problem. An effective way to reduce these third-party risks is to avoid high-risk areas in path planning, but most current path planning methods are optimized to minimize flight distance, and third-party risk costs are rarely considered. In this paper, a comprehensive risk-cost UAV path planning method is proposed, which evaluates urban risk by establishing a third-party risk model that incorporates obstacle risk, death risk, and property loss risk. This paper proposes a Min-cost A* algorithm based on city risk assessment, and smooths the generated low-risk path through the improved Floyd algorithm. The results show that the path planning method can effectively reduce the risk in the flight path, improve the reliability of the UAV flight path in the urban environment, and solve the problem of planning the third-party risk path of the UAV.

## Introduction

UAVs are a core component of urban air mobility^1,2^ and play an important role in smart city initiatives. As one of the key factors in autonomous flight, UAV path planning includes important links such as environmental modeling, path search, and path smoothing^3^. Common UAV path planning studies include the study of minimizing operational risks^4^, minimizing operating costs^5^, modeling the airspace environment, and optimizing the UAV path planning algorithm^6^.

For the problem of risk assessment in unmanned aerial vehicle (UAV) operations, researchers have proposed a UAV falling death probability calculation model, which effectively evaluates the risk of UAVs to road network safety by introducing factors such as spatial occlusion, accident occurrence probability, and risk exposure time^7,8^. In subsequent studies, researchers have provided data on different levels of damage caused by UAVs falling and impacting the human head, considering different heights and masses^9^. These data provide a basis for assessing the risks posed by UAV operations to ground personnel. Based on the classification of failure causes and failure modes in UAV operations, a risk assessment model based on Bayesian networks has been established^10^. This model can calculate the probabilities of ground collision accidents and intermediate events under different operating conditions. As the research progresses, researchers have proposed a UAV operating risk assessment model in urban environments, where collision probability is used to quantify each risk cost. They have addressed the trade-off between path integral risk measures and classical path efficiency by defining the problem of minimizing operational risk for multiple UAVs in partially unknown environments within a multi-criteria optimization framework^11,12^. Although the aforementioned studies have assessed the risks that UAVs may pose during flight, the evaluation models tend to focus on specific risks and have not established a comprehensive model for the integrated assessment of multiple risks.

In recent years, third-party risk analysis and modeling of UAVs have become a hot research topic. The personnel on board the aircraft (crew members and passengers) and those working within the airport are referred to as the first and second parties, respectively^13^. By introducing social and individual risk indicators, third-party risk is defined as safety concerns and property loss involving individuals or entities other than the UAV itself^14,15^. Assessing third-party risks is significant in UAV path planning for evaluating path risks. Researchers have planned cost-effective paths by analyzing risk factors associated with pedestrians, vehicles, and manned aircraft in urban environments^16^. Currently, factors such as obstacles^17^, weather conditions^18^, and other vehicles^19^ are considered to be third-party risk factors affecting UAV path planning. However, most studies only consider a single third-party risk and do not take into account the UAV’s own performance, leading to a singularized risk assessment and overly idealized path planning. To address this issue, this study extends third-party risk to include obstacle risk, fatality risk, and property loss risk in the risk modeling. A comprehensive UAV path planning model that considers third-party risks and incorporates the UAV’s own performance characteristics (such as bank angle, climb angle, minimum turning radius, minimum step size, flight altitude, and endurance) is proposed, with the UAV’s performance serving as constraints for path planning.

For different risk models and optimization goals, the researchers propose different UAV path planning algorithms, such as the A* algorithm^20^, the Dijkstra algorithm^21^, Particle Swarm Optimization (PSO)^22^, the Genetic algorithm (GA)^23^, etc. Researchers have utilized a risk assessment model to generate a risk-cost map. They have employed the Dijkstra algorithm, an enhanced version of the A* algorithm, and an improved Ant Colony Optimization (ACO) algorithm to generate UAV risk-cost–benefit paths^11^. However, the paths generated by these algorithms are often overly convoluted, making them challenging to apply in actual UAV flight scenarios. Subsequent research endeavors have commonly integrated UAV path-planning algorithms with path-smoothing techniques. For instance, researchers have combined the A* algorithm with Dubins curves to enhance the smoothness of UAV flight paths^24,25^. Nevertheless, these algorithms have primarily focused on either minimizing path cost or improving path smoothness, without concurrently addressing both minimal risk and path smoothness concerns in UAV path planning. In this study, an enhanced UAV path planning algorithm is proposed by modifying the traditional A* algorithm to consider third-party risks and integrated risk-cost analysis. The algorithm incorporates calculations of third-party risk costs to ensure minimal path risk. Additionally, an improved Floyd algorithm is employed to enhance the smoothness of the path while maintaining low-risk levels. The main contributions of this paper are as follows.

1. In response to the issue of singular risk assessment in UAV path planning, this paper, in the context of risk modeling and assessment, extends the concept of third-party risks to encompass obstacle risks, fatality risks, and property loss risks, and provides corresponding methods for evaluating these risks.
2. To address the issue of considering only a singular third-party risk in UAV path planning while neglecting the inherent capabilities of the UAV itself, this study proposes a novel path optimization method called UAV path planning based on third-party risk modeling. This method comprehensively takes into account third-party risks and incorporates the constraints of UAV's own performance, aiming to generate UAV paths that are more realistic and aligned with practical considerations.
3. In order to balance the minimization of path risk and the smoothness of the path in UAV path planning, this study proposes the Min-cost A* algorithm based on the A* algorithm. Additionally, by improving the Floyd algorithm, the proposed approach achieves a smoother path while minimizing the path risk.

The remainder of the paper is organized as follows. Chapter 2 discusses social indicators of third-party risk in an urban environment; Chapter 3 provides a detailed introduction to third-party risk modeling and assessment; Chapter 4 introduces the comprehensive risk cost UAV path planning model considering third-party risks and gives the specific solution algorithm. Chapter 5 conducts algorithm simulation and result analysis; Chapter 6 summarizes the findings of this paper.

## Background of the problem

### UAV description

A drone, or Unmanned Aerial Vehicle (UAV), is an aircraft capable of flying without the direct control of a human operator. Drones can be categorized into several types, including fixed-wing UAVs, multirotor UAVs, and single-rotor UAVs. Multirotor UAVs offer advantages such as vertical takeoff and landing capabilities, stable hovering, maneuverability, and payload-carrying capacity. These advantages have led to the widespread use of multirotor UAVs in various fields, including smart cities, aerial photography, surveillance, reconnaissance, and search and rescue operations. Therefore, this paper focuses on the study of quadcopter UAVs, a type of multirotor UAV.

### Risk factors in urban environment

**Figure 1:**
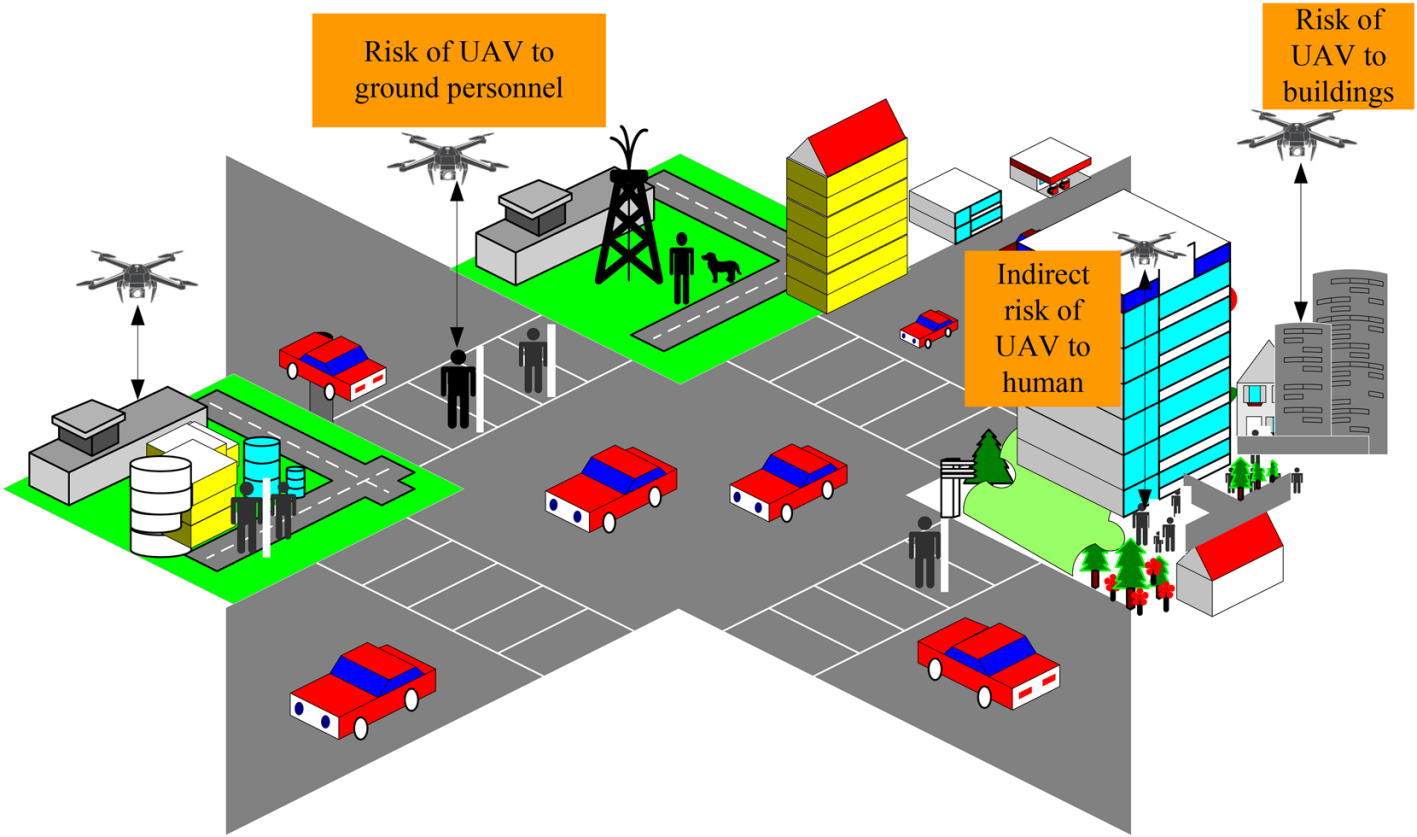
Schematic diagram of urban environmental risk factors. In the figure, the main risks associated with drones include the risk to ground personnel, the risk to buildings, and the risk of direct fatalities caused by drones.

The types of drone risks vary depending on the operating environment. For example, in a factory environment, the main risks are the risk of obstacles^26^, and in residential areas are mainly risk of crowd density, vehicle density, and noise impact^27,28^. In urban environments, the flight risks of drones include the risks to ground vehicles^19,29^, risks to ground personnel^14,15^, risks of collision between small UAV and manned UAVs in the air^30–32^, risks of UAVs operation^34^, noise effects^33^, etc. As shown in Fig. 1, in the urban environment, the population, buildings, and other space obstacles are the main risk factors affecting the operation of drones.

**Figure 2:**
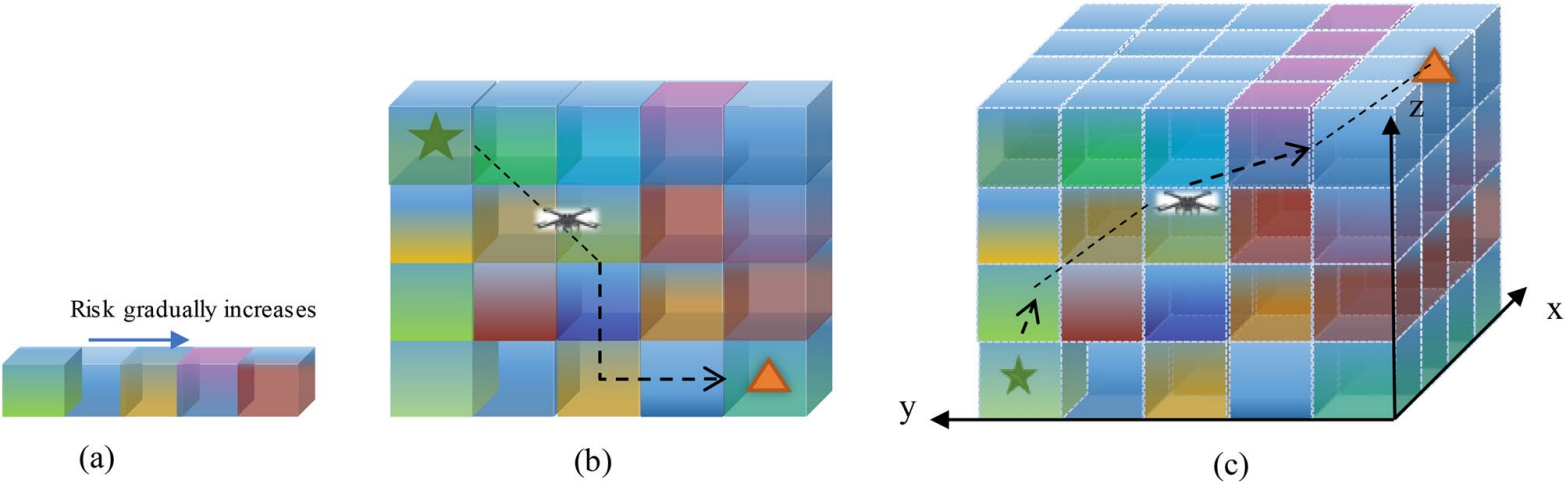
Schematic diagram of the division of low-altitude urban airspace. Note: (**a**) The different colors of the air block represent different degrees of risk, the brighter the color, the higher the risk; (**b**) indicates that the drone flight path in the risk air block unit will avoid the high-risk gas block; (**c**) is a schematic diagram of the drone flight path in a three-dimensional risk environment.

For the urban operation environment, this article mainly studies the following risks:

1. Obstacle risk: the risk of drones in the operation of urban environments due to obstacles such as office buildings and residential buildings;
2. Death risk: drones hit pedestrians and buildings on the ground, causing casualties;
3. Property loss risk: UAV hitting urban infrastructure or high-rise buildings during flight and causing property damage;

In urban environments, people, buildings, and obstacles are distributed discretely. To quantitatively evaluate risks in the urban operating environment, as shown in Fig. 2, this document divides the low-altitude urban airspace into discrete three-dimensional gas block units and performs UAV path planning based on the above risks.

### Symbol definitions

This study investigates the comprehensive risk-cost path planning of drones considering third-party risks. In the examination and modeling of the problem, multiple equations and symbols were employed. Table 1 presents the definitions and explanations of all the symbols used in this study, elaborating their respective meanings.

**Table 1:** Symbol definitions in this study.

Terms	Definitions and descriptions	Terms	Definitions and descriptions
$O$	Obstacle risk	$P$	Property Loss Risk
${V}_{obstacle}\left(r\right)$	The volume of obstacles in a three-dimensional gas block cell with side length $r$	${V}_{surround}\left(r\right)$	The volume of a three-dimensional gas block element with side length $r$
$D$	Death risk	$\xi$	The skin penetration degree
${D}_{1}$	Direct death risk	${r}_{UAV}$	The radius of the drone
${D}_{2}$	Indirect death risk	${r}_{Human}$	The radius of the person
${P}_{crash}$	The probability that the drone fails to fall	${P}_{impact}$	The probability that the drone falls to hit people
${N}_{hit}^{p}$	The number of people hit by the drone	${P}_{D}^{p}$	The mortality rate in the drone accident
${E}_{imp}$	The impact kinetic energy	${s}_{c}$	The occlusion factor
${\sigma }_{p}$	The density of the population	${\sigma }_{b}$	The density of the building
${N}_{D}^{p}$	The average number of casualties caused by a drone colliding with a building	${A}_{P}$	The contact area (A_P) of the crash between the falling drone and the person
${N}_{b\_impact}$	The number of buildings impacted by the drone's fall	${S}_{hit}$	The area expected to be hit by the drone
${S}_{b\_hit}$	The estimated drone impact area	$m$	The mass of the drone
$\Delta h$	The change of the drone flight altitude	${\alpha }_{1,2,3}$	The weight factor of Obstacle risk, death risk and Property risk
${\varphi }_{max}$	The maximum steering angle of the drone	${\theta }_{max}$	The maximum climb angle of the drone
${r}_{min}$	The limit turning radius	${v}_{min}$	The minimum flight speed
${n}_{ymax}$	Indicates the maximum normal overload of the drone	${t}_{max}$	The maximum UAV endurance time
${H}_{min}$	The minimum flight altitude	${H}_{max}$	The maximum flight altitude
$l$	The minimum step length	$g$	Gravity acceleration
$f\left(n\right)$	The comprehensive priority of node $n$,	$g\left(n\right)$	The cost of the node $n$ from the starting point
$h\left(n\right)$	The estimated cost of node n from the endpoint	${G}_{cost}\left(n\right)$	The cost function of the Min-cost A* algorithm
${H}_{cost}\left(n\right)$	The heuristic function of the Min-cost A* algorithm	${H}_{cost}^{*}\left(n\right)$	The optimized heuristic function
${H}_{cost}^{*}\left(n\right)$	The optimized heuristic function	$d\left(n\right)$	The Euclidean distance
$A$	The Manhattan distance from the current node to the endpoint	$B$	The Manhattan distance from the current node to the starting point
${\text{w}}$	Judgment matrix	C	Feature matrix
$C.I.$	The consistency index	$R.I.$	The random index
$C.R.$	The consistency ratio	${w}^{0}$	The relative weight
${\uplambda }_{{\text{max}}}$	The maximum eigenvalue of the judgment matrix	${a}_{ij}$	The result of comparison between elements $i$ and $j$

These symbols are distributed across the following three sections: (1) Third-Party Risk Modeling and Assessment, (2) UAV Path Planning Modeling, and (3) Algorithmic Solutions.

## Third-party risk modeling and assessment

### Obstacle risk model

**Figure 3:**
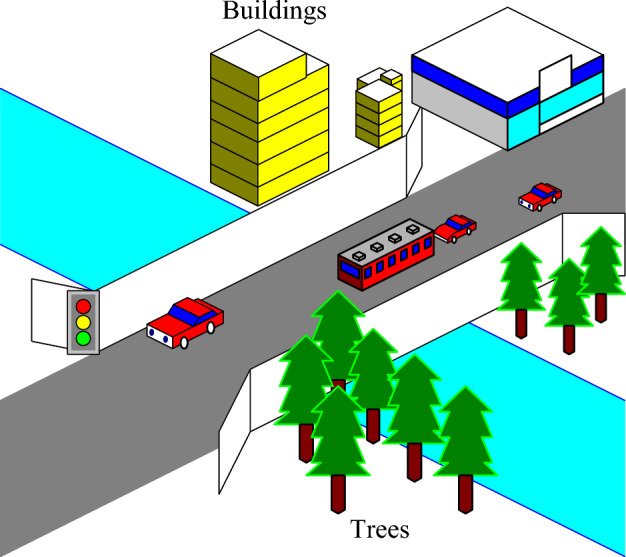
Schematic diagram of urban obstacles. In urban environments, physical obstacles mainly consist of buildings, trees, power grids, and so on.

Physical barriers in cities include static objects such as buildings, power grids, and trees, as shown in Fig. 3. UAVs have a higher probability of touching obstacles during climbing, turning, and other actions. Therefore, obstacle risk is considered the fundamental risk type in three-dimensional path planning.

In UAV path planning, common methods for assessing obstacles include grid maps and probabilistic maps. Grid maps partition the environment into regular grids and assign a state (occupied or unoccupied) to each grid cell, enabling fast collision detection. However, the grid resolution can affect map precision and computational complexity, leading to quantization errors. Probabilistic maps, on the other hand, use probability models to represent the likelihood of obstacle presence at each location in the environment. Common types of probabilistic maps include occupancy grid maps and Gaussian mixture models. This approach requires substantial storage and computational resources, potentially limiting real-time data updates and fusion. In consideration of obstacle assessment errors, computational and storage resources, and overall costs, this study introduces “risk radius $\left(r\right)$” to evaluate obstacle risks. Within the three-dimensional atmospheric blocks in urban airspace (Fig. 2), we assess the risks associated with obstacles within these atmospheric block units. The assessment is based on the ratio of the volume of obstacles within a three-dimensional block unit with a side length of $r$ to the volume of the three-dimensional block unit itself. This approach enables a holistic evaluation of spatial obstacle risks. Relative to grid maps, this method circumvents quantization errors stemming from grid resolution. Simultaneously, it avoids the constraints on data updates and fusion imposed by probabilistic maps due to probabilistic inference. This results in algorithmic time savings, facilitating the rapid planning of flight paths. Obstacle risk $O$ is recorded as:

1 $$O=\frac{{V}_{obstacle}\left(r\right)}{{V}_{surround}\left(r\right)}$$

${V}_{obstacle}\left(r\right)$ represents the volume of obstacles in a three-dimensional gas block cell with side length $r$ and ${V}_{surround}\left(r\right)$ represents the volume of a three-dimensional gas block element with side length $r$.

### Death risk model

Cities are densely populated, and effective assessment of death risk is a safety prerequisite for drone path planning. Hu^19^ and Izdebski^29^ defined and evaluated the risk of death in different situations, which improved the safety rate of the terrestrial population. In most studies, the assessment of mortality risk pertains primarily to the evaluation of direct death risk, which refers to the assessment of casualties resulting directly from a UAV's fall. This paper introduces an evaluation of casualties resulting from indirect death risk, encompassing the risk associated with a drone colliding with either a crowd or a structure. The death risk ($D$) is defined as the combined result of the direct death risk ${D}_{1}$ and the indirect death risk ${D}_{2}$. This comprehensive approach aims to provide a more thorough assessment of the death risk posed by drones operating in urban environments.

2 $$D={D}_{1}+{D}_{2}$$

#### Direct death risk

**Figure 4:**
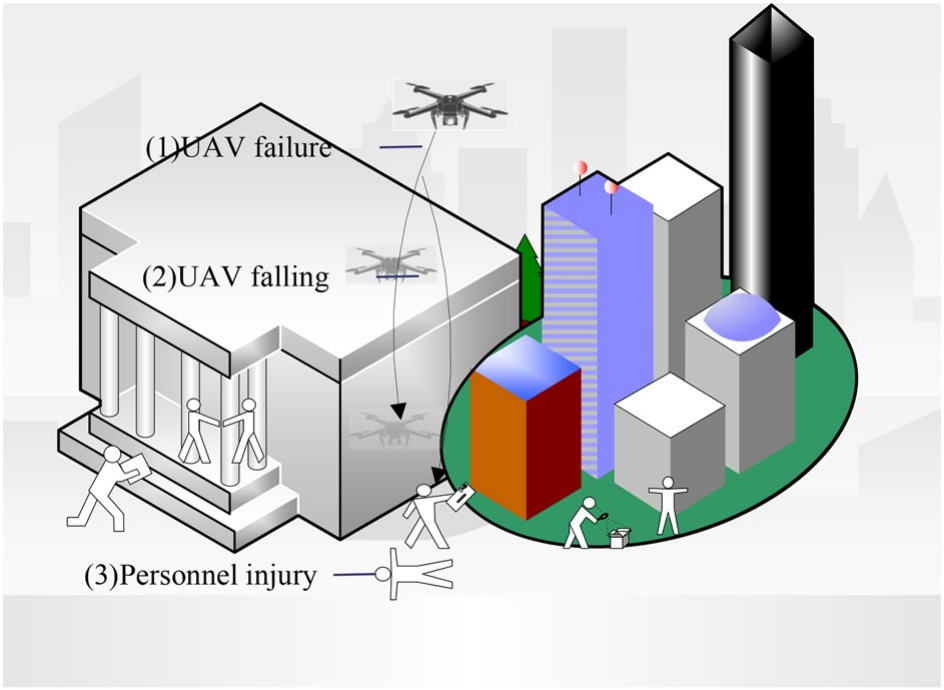
The drone crash stage division. The process of a drone falling can be divided into three stages: system failure, drone descent, and injury to personnel caused by the descent.

During the flight process, a drone may lose control and experience a fall due to factors such as navigation failure, low battery power, program malfunction, and other reasons. Under normal circumstances, the drone accident can be divided into three stages, as shown in Fig. 4: (1) the drone fails during flight and cannot fly normally; (2) the fault cannot be repaired in time and the drone falls; (3) the falling drone causes damage to personnel on the ground.

Pang^4^ combined the three stages of drone fall to establish a pedestrian death risk model. This model integrates the probability of drone system failure and descent, the number of individuals hit by the drone, and the probability of fatality after a drone collision to evaluate the death risk. However, this model does not take into account the probability of a drone striking a person during descent. In the context of analyzing the drone descent process, when a drone experiences an accident and enters the third stage (where the falling drone causes injuries to individuals on the ground), this event can be considered an independent occurrence. In other words, the incidence of each descent event does not have a mutual influence on one another. Therefore, the number of fatalities resulting from each descent event can be expressed as the product of the total number of individuals within the drone descent area and the probability of a drone striking a person during descent and subsequently causing a fatality. The probability of a drone striking a person during descent and causing a fatality is equal to the product of probability of a drone system failure and descent, a drone striking a person during descent, and a fatality occurring following a drone-person collision. We designate the direct fatality risk resulting from each descent event as ${D}_{1}$, expressed as:

3 $${D}_{1}={{N}_{hit}^{p}P}_{crash}{P}_{impact}{P}_{D}^{p}$$

where ${P}_{crash}$ represents the probability that the drone fails to fall, ${P}_{impact}$ represents the probability that the drone falls to hit people, ${N}_{hit}^{p}$ represents the number of people hit by the drone and ${P}_{D}^{p}$ represents the probability of causing human death after a drone fall impacts a person.

${P}_{crash}$ is determined by the reliability of the hardware and software of the unmanned aerial system. According to the research of the Joint Regulatory Organization for Unmanned Aerial Vehicles, $1\times {10}^{-4}$ per flight hour (pfh) is used as the minimum accident rate of RPAS (remotely piloted aircraft system), from which the probability of accident can be calculated^9^, this article $1\times {10}^{-6}$ is used as the calculation value of the ${P}_{crash}$.

${P}_{D}^{p}$ is related to impact kinetic energy, space shielding factors, etc., and this article uses the method proposed by Dalamagkidis^35^ to calculate the probability of death from the accident ${P}_{D}^{p}$:

4 $${P}_{D}^{p}=\frac{1}{1+\sqrt{\frac{\alpha }{\beta }{\left(\frac{\beta }{{E}_{imp}}\right)}^{\frac{1}{{4s}_{c}}}}}$$

where ${E}_{imp}$ represents the impact kinetic energy and ${{\varvec{s}}}_{{\varvec{c}}}$ represents the occlusion factor, which is an absolute real number in the range of (0,1]^20^. The average value of the ${{\varvec{s}}}_{{\varvec{c}}}$ is 0.5, representing 50% probability of the shock energy causing death, $\beta$ is the threshold of the shock energy required to cause death when the ${{\varvec{s}}}_{{\varvec{c}}}$ tends to zero^7^. Based on the above research, this article takes $\alpha$ =106 J, $\beta$ =100 J.

**Figure 5:**
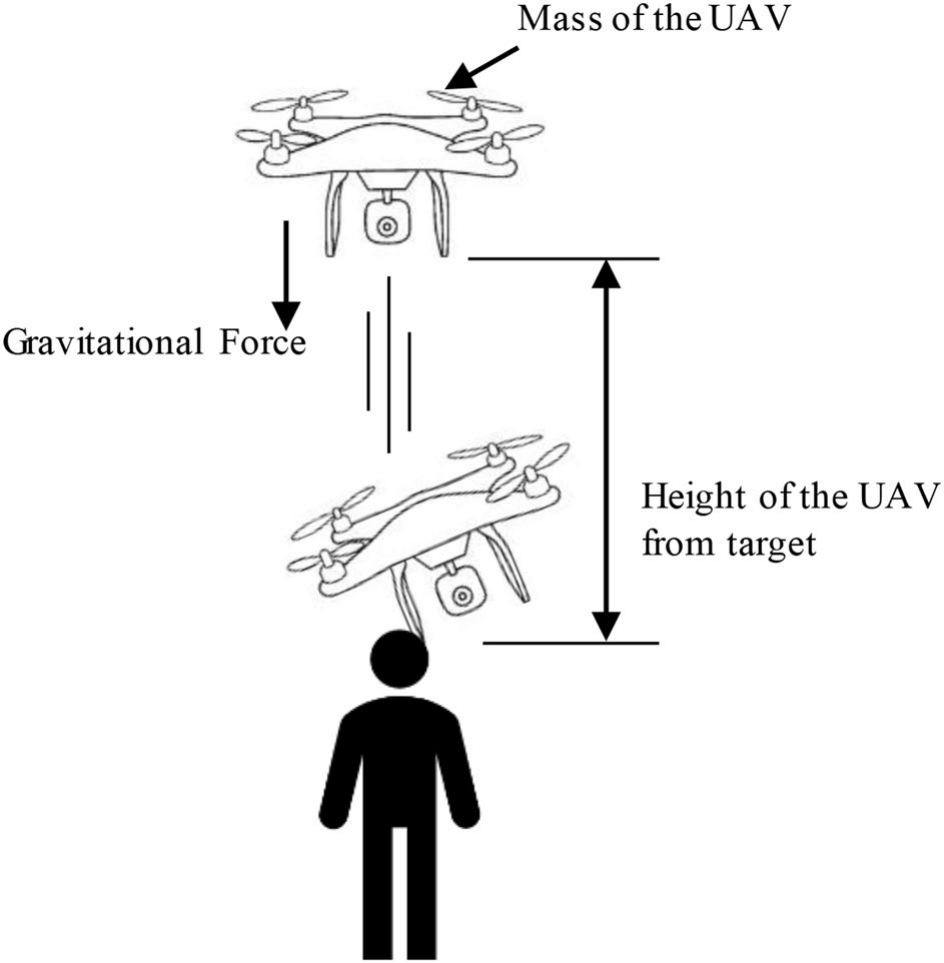
Schematic diagram of drone falling from height of h. The descent of a drone with a certain weight from a height h in the air generates a certain amount of kinetic energy, which is one of the factors influencing the fatality risk.

This paper combines the physical equations of kinetic energy to calculate the ${E}_{imp}$, as shown in Fig. 5, the impact kinetic energy generated by the fall of the drone is related to the mass of the drone itself and the instantaneous landing speed, which is denoted as:

5 $${ E}_{imp}=\frac{1}{2}m{v}^{2}$$

where $m$ (kg) is the mass of the falling drone and $v$ is the instantaneous speed of the drone when it lands.

In Eq. (3), the number of people hit by the drone (${N}_{hit}^{p}$) is related to the density of the ground population (${\sigma }_{p}$) and the area (${S}_{hit}$) expected to be hit by the drone, which is denoted as:

6 $${N}_{hit}^{p}={S}_{hit}{\sigma }_{p}$$

The expected impact area can be estimated based on the radius of the drone and the radius of the person, which can be expressed as:

7 $${S}_{hit}=\pi {\left({r}_{UAV}+{r}_{Human}\right)}^{2}$$

where ${r}_{UAV}$ indicates the radius of the drone, depending on the drone model, ${r}_{Human}$ represents the radius of the person, and the ${r}_{Human}$ in this study is 0.164 m.

${P}_{impact}$ is an important factor in the casualties caused by unmanned falls, it reflects the probability that a UAV falls to hit people, and the introduction of ${P}_{impact}$ can make the death risk assessment closer to the actual situation. Usually, ${P}_{impact}$ is related to the density of the population on the ground (${\sigma }_{p}$) and the contact area (${A}_{P}$) of the crash between the falling drone and the person, this paper adds the skin penetration degree coefficient ($\xi$) based on it. Skin penetration occurs at high kinetic energy density (high energy in a small impact area), and when the drone falls, the carbon fiber frame can cause varying degrees of damage to human skin, which is the direct cause of casualties. Therefore, the introduction of the skin penetration coefficient into the evaluation of the ${P}_{impact}$ can improve the accuracy of the assessment results, ${P}_{impact}$ is recorded as:

8 $${P}_{impact}={\xi \sigma }_{p}{A}_{P}$$

During fall, the drone can hit the head, chest, or abdomen, slightly increasing the value of the ${A}_{P}$, and the ${A}_{P}$ is between 0.25 and 0.6^36^. $\xi$ is an absolute real number whose range is (0,1], the greater value of $\xi$, the higher the degree of penetration of the skin.

From this analysis, the direct death risk model is as follows:

9 $$\left\{\begin{array}{l}{D}_{1}={P}_{crash}{P}_{impact}{P}_{D}^{p}{N}_{hit}^{p}\\ {P}_{impact}={\xi \sigma }_{p}{A}_{P}\\ { N}_{hit}^{p}={S}_{hit}{\sigma }_{p}\\ {P}_{D}^{p}=\frac{1}{1+\sqrt{\frac{\alpha }{\beta }{\left(\frac{\beta }{{E}_{imp}}\right)}^{\frac{1}{{4s}_{c}}}}}\\ {S}_{hit}=\pi {\left({r}_{UAV}+{r}_{Human}\right)}^{2}\end{array}\right.$$

#### Indirect death risk

Drone falls hitting buildings can cause casualties; this paper combines the density of buildings for indirect death risk assessment. The risk of indirect death is ${D}_{2}$, and the main difference between ${D}_{1}$ and ${D}_{2}$ is that the former emphasizes that the casualties caused by the direct impact of the drone fall are related to the population density, the latter is the indirect casualties caused by drones hitting buildings which related to the density of buildings on the ground, ${D}_{2}$ is recorded as:

10 $${D}_{2}={P}_{crash}{N}_{b\_impact}{N}_{D}^{p}$$

where ${P}_{crash}$ indicates the probability of a drone failure to fall, ${N}_{b\_impact}$ indicates the number of buildings impacted by the drone’s fall, and ${N}_{D}^{p}$ indicates the average number of casualties caused by a drone colliding with a building, and its value is 0.75^37^

${N}_{b\_impact}$ is determined by the density of the building (${\sigma }_{b}$) and estimated drone impact area(${S}_{b\_hit}$) , which is denoted as:

11 $${N}_{b\_impact}={\sigma }_{b}{S}_{b\_hit}$$

where ${S}_{b\_hit}$ indicates the estimated drone impact area, and here is the formula for calculating the ${S}_{b\_hit}$:

12 $${S}_{b\_hit}=\pi {\left({r}_{UAV}\right)}^{2}$$

The indirect death risk assessment model are:

13 $$\left\{\begin{array}{l}{D}_{2}={P}_{crash}{N}_{b\_impact}{N}_{D}^{p}\\ { N}_{hit}^{b}={\sigma }_{b}{S}_{b\_hit}\\ {S}_{b\_hit}=\pi {\left({r}_{UAV}\right)}^{2}\end{array}\right.$$

### Property loss risk model

**Figure 6:**
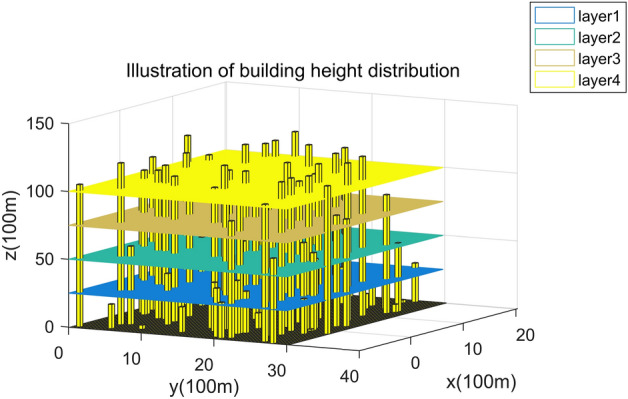
Changes in building height. In urban environments, unevenly spaced buildings can impact the safety of drone flights. Drone crashes and collisions can result in property damage.

Property loss risk refers to the potential losses that may occur when a drone collides with buildings or public infrastructure during its flight, as shown in Fig. 6. Firstly, the magnitude of damage caused by the collision is related to the drone's flight altitude, with higher altitudes resulting in greater energy during a fall and consequently more severe property damage. Secondly, the extent of damage caused by the collision is also dependent on the kinetic energy of the drone during flight, with higher flight speeds leading to greater kinetic energy and the potential for more substantial property damage. This paper assesses property damage caused by variations in drone altitude and flight speed. Property damage risk is defined as follows:

14 $$P=mg\Delta h+\frac{1}{2}m{v}^{2}$$

where $v$ is the speed of the drone during flight, $m$ is the mass of the drone, $\Delta h$ is the change of the drone flight altitude, and $g$ takes 9.8 $m/{s}^{2}$.

## Planning of the UAV path based on risk cost

To solve the problem of risk-based drone path planning, this article establishes a UAV path planning model that contains obstacle risk, death risk, and property loss risk. We also propose a new A* algorithm named Min-cost A* algorithm. Finally, we improve the Floyd algorithm and use it to smooth the path.

### UAV path-planning model based on third-party risk

The UAV path planning problem with comprehensive risk cost can be regarded as a non-linear optimization problem, and the decision variables are the third-party risks (obstacle risk, death risk, property loss risk) established. The objective function is recorded as:

15 $${\text{min}}\,Z={\alpha }_{1}O{+\alpha }_{2}D+{\alpha }_{3}P$$

where $O$ represents the cost of obstacles, $D$ represents the death risk, $P$ represents the property damage risk, ${\alpha }_{1}, {\alpha }_{2}, {\alpha }_{3}$ represents the weight factor of each risk, and ${\alpha }_{1}{+\alpha }_{2}+{\alpha }_{3}=1$.

In this paper, the relevant performance of the UAV, such as steering angle, climb angle, limit turning radius, flight altitude, endurance time, and minimum step size, are used as constraints to improve the reliability of UAV flight.

Constraints on UAV steering angle:

16 $$arccos\left[\frac{\left({x}_{i}-{x}_{i-1}\right)\left({x}_{i+1}-{x}_{i}\right)+\left({y}_{i}-{y}_{i-1}\right)\left({y}_{i+1}-{y}_{i}\right)}{\sqrt{{\left({x}_{i}-{x}_{i-1}\right)}^{2}+{\left({y}_{i}-{y}_{i-1}\right)}^{2}}\sqrt{{\left({x}_{i+1}-{x}_{i}\right)}^{2}+{\left({y}_{i+1}-{y}_{i}\right)}^{2}}}\right]\ge 0$$

17 $$arccos\left[\frac{\left({x}_{i}-{x}_{i-1}\right)\left({x}_{i+1}-{x}_{i}\right)+\left({y}_{i}-{y}_{i-1}\right)\left({y}_{i+1}-{y}_{i}\right)}{\sqrt{{\left({x}_{i}-{x}_{i-1}\right)}^{2}+{\left({y}_{i}-{y}_{i-1}\right)}^{2}}\sqrt{{\left({x}_{i+1}-{x}_{i}\right)}^{2}+{\left({y}_{i+1}-{y}_{i}\right)}^{2}}}\right]\le {\varphi }_{max}$$

where ${\varphi }_{max}$ represents the maximum steering angle of the drone, $\left({x}_{i}, {y}_{i},{z}_{i}\right)$ represents the current location of the UAV node, $\left({x}_{i+1}, {y}_{i+1}, {z}_{i+1}\right)$ represents the next location of the UAV node, and $\left({x}_{i-1}, {y}_{i-1}, {z}_{i-1}\right)$ represents the previous location of the UAV node.

Constraints on UAV climbing angle:

18 $$0\le {\theta }_{i}=\frac{\left|{z}_{i}-{z}_{i-1}\right|}{\sqrt{{\left({x}_{i}-{x}_{i-1}\right)}^{2}+{\left({y}_{i}-{y}_{i-1}\right)}^{2}}}\le {\text{tan}}{\theta }_{max}$$

where ${\theta }_{max}$ represents the maximum climb angle of the drone, and ${\theta }_{i}$ is the climbing angle of the current position.

Constraints on UAV turning radius:

19 $${r}_{i}\ge {r}_{min}=\frac{({{v}_{min})}^{2}}{g\sqrt{{{(n}_{ymax})}^{2}}}$$

where ${r}_{i}$ indicates the turning radius of the current position of the drone, ${r}_{min}$ indicates the limit turning radius, ${v}_{min}$ indicates the minimum flight speed, and ${n}_{ymax}$ indicates the maximum normal overload of the drone.

In urban environments, the flight altitude of UAVs is not only related to the performance of UAVs themselves, but also limited by the urban UAV flight management system, and the UAV flight altitude constraints are as follows:

20 $${H}_{min}\le {h}_{i}\le {H}_{max}$$

Among them ${h}_{i}$ indicates the current flight altitude of the drone and ${H}_{min}$ and ${H}_{max}$ represent the minimum flight altitude and maximum flight altitude of the drone restricted by the city.

The endurance time of the drone is affected by the battery level, so the flight time should be strictly less than the endurance time, and the flight time constraints of the drone are as follows:

21 $$\sum_{i=1}^{n}{t}_{i}<{t}_{max}$$

where $\sum_{i=1}^{n}{t}_{i}$ indicates the total UAV flight time from the departure point to the current location, and ${t}_{max}$ indicates the maximum UAV endurance time.

In the actual flight of the drone, the constraint of the shortest distance that must fly directly before changing the flight attitude is called the minimum step length constraint, which can improve the reliability of the UAV attitude adjustment process, and the minimum step length constraint as follows:

22 $${l}_{i}\ge l$$

Among them, ${l}_{i}$ indicates the step length of the drone to make attitude adjustment in the current position, and $l$ represents the minimum step length.

In summary, the risk-based cost UAV path planning model can be expressed as follows:

$${\text{min}}\,Z={\alpha }_{1}O{+\alpha }_{2}D+{\alpha }_{3}P$$

23 $$s.t\left\{\begin{array}{l}arccos\left[\frac{\left({x}_{i}-{x}_{i-1}\right)\left({x}_{i+1}-{x}_{i}\right)+\left({y}_{i}-{y}_{i-1}\right)\left({y}_{i+1}-{y}_{i}\right)}{\sqrt{{\left({x}_{i}-{x}_{i-1}\right)}^{2}+{\left({y}_{i}-{y}_{i-1}\right)}^{2}}\sqrt{{\left({x}_{i+1}-{x}_{i}\right)}^{2}+{\left({y}_{i+1}-{y}_{i}\right)}^{2}}}\right]\ge 0\\ arccos\left[\frac{\left({x}_{i}-{x}_{i-1}\right)\left({x}_{i+1}-{x}_{i}\right)+\left({y}_{i}-{y}_{i-1}\right)\left({y}_{i+1}-{y}_{i}\right)}{\sqrt{{\left({x}_{i}-{x}_{i-1}\right)}^{2}+{\left({y}_{i}-{y}_{i-1}\right)}^{2}}\sqrt{{\left({x}_{i+1}-{x}_{i}\right)}^{2}+{\left({y}_{i+1}-{y}_{i}\right)}^{2}}}\right]\le {\varphi }_{max}\\ 0\le {\theta }_{i}=\frac{\left|{z}_{i}-{z}_{i-1}\right|}{\sqrt{{\left({x}_{i}-{x}_{i-1}\right)}^{2}+{\left({y}_{i}-{y}_{i-1}\right)}^{2}}}\le {\text{tan}}\,{\theta }_{max} \\ {r}_{i}\ge {r}_{min}=\frac{({{v}_{min})}^{2}}{g\sqrt{{{(n}_{ymax})}^{2}}}\\ {H}_{min}\le {h}_{i}\le {H}_{max}\\ \sum_{i=1}^{n}{t}_{i}<{t}_{max}\\ {l}_{i}\ge l\end{array}\right.$$

### Path-planning solution algorithm based on risk cost

This section details path planning solutions based on risk costs, that is, the Min-cost A* algorithm. The algorithm retains the characteristics of the A* algorithm that responds quickly to the environment, and we improve the cost function and heuristic function accordingly to improve the accuracy and speed of the algorithm. We also calculate the weight factors of risks by the analytic hierarchy method and smooth the risk path by the improved Floyd algorithm.

#### Min-cost A*algorithm

The A* algorithm is a heuristic algorithm, the basic idea is that when the drone starts to run, use the environmental prior information to calculate the cost between points, the algorithm marks the selected points as a set M, and the unselected point set is recorded as N. At the beginning of the algorithm, only the initial point exists in the set M, and N contains other nodes except the starting point, select the path point with the lowest cost to join the set M, and then update the information from the M and N set. Continue selecting the point in N that costs the least to reach the point in M until the shortest path is obtained.

**Figure 7:**
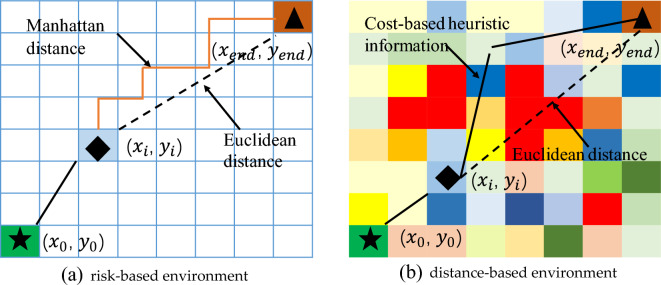
Heuristic values in different environments. Note: (**a**) indicates that in distance-based environment, Manhattan distance and Euclidean distance are used as the traditional A* algorithm heuristic distance. (**b**) Indicates that in a cost-based environment, the uneven distribution of risk costs from the current node (${x}_{i}$, ${y}_{i}$) to the target node $({{\text{x}}}_{{\text{end}}}$, ${y}_{end}$) makes it difficult for the algorithm to select the heuristic function.

Equation (24) is the node priority calculation method of the A* algorithm. During execution of the algorithm, the node with the smallest value of $f\left(n\right)$ will be the next node to search.

24 $$f\left(n\right)=g\left(n\right)+h\left(n\right)$$

where $f\left(n\right)$ is the comprehensive priority of node $n$, $g\left(n\right)$ is the cost of the node $n$ from the starting point, and $h\left(n\right)$ is the estimated cost of node n from the endpoint, that is, the heuristic function of the A* algorithm. A commonly used heuristic function is to represent the cost in terms of distance, that is, the shortest distance. In distance-based environments, commonly used distances are Manhattan distance or Euclidean distance, as shown in Fig. 7a. However, in a risk-based environment, as shown in Fig. 7b, the distribution of risk cost values in different spatial units is uneven and unpredictable, and it is difficult to obtain suitable heuristic values, but $h\left(n\right)$ is critical to the accuracy and speed of the algorithm.

The A* algorithm must be able to find the shortest path when $h\left(n\right)$ is always less than or equal to the cost of the node $n$ to the endpoint. But in this case, the number of nodes searched by the algorithm increases, and the speed of the algorithm decreases. The A* algorithm can quickly find the optimal path when $h\left(n\right)$ is equal to the cost from node $n$ to the endpoint. However, it is difficult to achieve this effect in the actual situation because it is difficult to calculate how far the distance is before the end. If the value of $h\left(n\right)$ is more expensive than the node $n$ to the endpoint, the A* algorithm is unlikely to find the shortest path, but the algorithm is fast. In extreme cases, when the heuristic function $h\left(n\right)$ is always 0, $g\left(n\right)$ will determine the priority of the node, which is the Dijkstra algorithm. At the other extreme, if $h\left(n\right)$ is much larger than $g\left(n\right)$, this is the best priority search.

Combined with the above analysis, aiming at the selection of the heuristic function in the risk-cost environment and the UAV path planning problem-based on risk cost, this paper improves the cost function and heuristic function of the traditional A* algorithm and proposes the Min-cost A* calculation, which is as follows.

Based on the minimum cost function $g\left(n\right)$ under the influence of multiple factors, the Min-cost A* algorithm avoids the problem that the traditional A* algorithm only calculates the flight range and adds third-party risk generation value. The cost function of the Min-cost A* algorithm is the objective function of the UAV path planning model based on risk cost, expressed as:

25 $${G}_{cost}\left(n\right)={\alpha }_{1}O{+\alpha }_{2}D+{\alpha }_{3}P$$

where ${G}_{cost}\left(n\right)$ represents the cost function of the Min-cost A* algorithm, $O$ represents the risk of obstacles, $D$ represents the risk of death, $P$ represents the risk of property damage, ${\alpha }_{1}+{\alpha }_{2}+{\alpha }_{3}=1$

The heuristic function of the Min-cost A* algorithm are as follows:

26 $${H}_{cost}\left(n\right)={\varphi }_{1}d\left(n\right)+{\varphi }_{2}{d\left(n\right){O}_{next}+\varphi }_{3}d\left(n\right){D}_{next}+{\varphi }_{4}d\left(n\right){P}_{next}$$

where ${\varphi }_{1}+{\varphi }_{2}+{\varphi }_{3}+{\varphi }_{4}=1$,${H}_{cost}\left(n\right)$ is the heuristic function after the first optimization of the Min-Cost A* algorithm, ${X}_{next}$ is the estimated risk cost of the next search node, $d\left(n\right)$ is the Euclidean distance from the current node to the endpoint, and the calculation formula is:

27 $$d\left(n\right)=\sqrt{{\left({x}_{i}-{x}_{end}\right)}^{2}+{\left({y}_{i}-{y}_{end}\right)}^{2}+{\left({z}_{i}-{z}_{end}\right)}^{2}}$$

where $\left({x}_{i}, {y}_{i}{,z}_{i}\right)$ represents the current search node and $\left({x}_{end}, {y}_{end}{, z}_{end}\right)$ represents the endpoint. In case the algorithm fails to find an optimal solution due to too large a heuristic value, we optimize the heuristic function again, and the optimization strategy is as follows:

28 $${H}_{cost}^{*}\left(n\right)=(1+\frac{A}{B}){H}_{cost}\left(n\right)$$

where ${H}_{cost}^{*}\left(n\right)$ represents the final heuristic function of the Min-Cost A* algorithm, $A$ represents the Manhattan distance from the current node $\left({x}_{i}l, {y}_{i}{, z}_{i}\right)$ to the endpoint $\left({x}_{end}, {y}_{end}{, z}_{end}\right)$ and $B$ is the Manhattan distance from the current node to the starting point $\left({x}_{start}, {y}_{start}{, z}_{start}\right)$. The calculation formula is:

29 $$\left\{\begin{array}{c}{B}_{i,start}=\left|{x}_{i}-{x}_{start}\right|+\left|{y}_{i}-{y}_{start}\right|+\left|{z}_{i}-{z}_{start}\right|\\ {A}_{i,end}=\left|{x}_{i}-{x}_{end}\right|+\left|{y}_{i}-{y}_{end}\right|+\left|{z}_{i}-{z}_{end}\right|\end{array}\right.$$

According to Eq. (28), the algorithm can be improved to set the adaptive heuristic function weights. The farther the distance from the current position to the endpoint, the more risk factors and the greater weight of the heuristic. Therefore, the combined cost of the Min-cost A* algorithm is expressed as:

30 $${f}_{cost}\left(n\right)={G}_{cost}\left(n\right)+(1+\frac{A}{B}){H}_{cost}^{*}\left(n\right)$$

**Figure 8:**
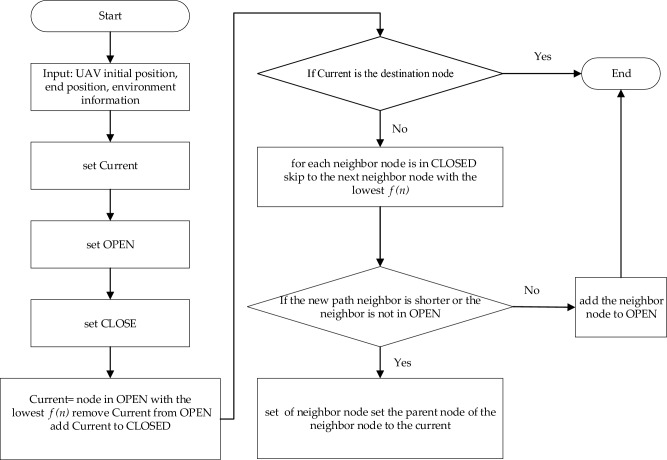
The pseudocode flowchart for the Min-cost A* algorithm. Note that Current represents Store’s current location (a collection), OPEN represents Storage low-risk nodes, CLOSED represents Storage non-optional nodes, high-risk cost nodes.

The Min-cost A* algorithm employs a greedy approach, selecting a locally optimal solution at each step to ensure that the final result approximates the global optimal solution. The algorithm uses a cost function based on the risk-cost UAV path planning model's objective function (as shown in Formula (25)). It searches for path nodes with the minimum risk, expanding in the order of increasing $f\left(n\right)$ values. This means that nodes with smaller $f\left(n\right)$ values are expanded first, thereby finding the optimal solution. The improved algorithm reduces search space, improves speed, and prevents the algorithm from falling into the local optimal caused by the large estimation of the heuristic function. The pseudocode of the program for the Min-cost A* algorithm is shown in Fig. 8.

#### Calculation of the weight of the cost function

**Figure 9:**
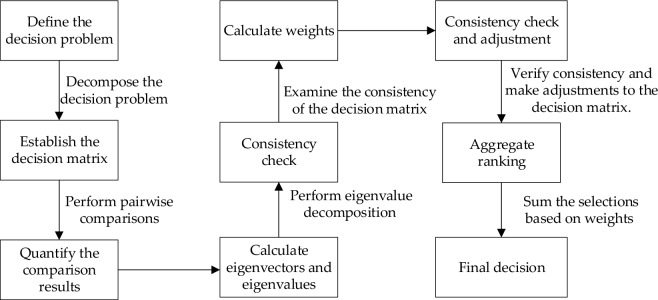
Process of using Analytic Hierarchy Process (AHP) to solve a flowchart. The complete solving steps are divided into the aforementioned 8 parts. This article uses this method to calculate the risk weights.

In Section “Min-cost A*algorithm”, the cost function selects the sum of the city's third-party risk costs, and the risk factor weights represent the degree of influence of different risks in UAV path planning. This study uses the Analytic Hierarchy Process (AHP) to determine the weights of risk factors. The general steps of the AHP method are shown in Fig. 9.

**Figure 10:**
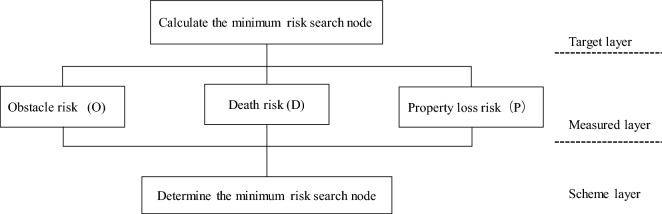
AHP hierarchy. Measured layer includes the Obstacle risk, Death risk, and Property loss risk.

The first step of the Analytic Hierarchy Process (AHP) is to identify the objectives and their hierarchical relationships. In our case, the objective is to determine the weight relationships between obstacles, risk of death, and property damage. Therefore, this article constructs a three-level hierarchy consisting of the goal level, criteria level, and alternative level, as shown in Fig. 10.

The second step involves constructing judgment matrices to determine the relative importance between criteria and sub-criteria at each level through pairwise comparisons. In this article, the authors use the consistency matrix method along with a scale of 9 importance levels (with higher numbers indicating higher relative importance, e.g., if A is 9 relative to B, then B is 1/9 relative to A) to establish the judgment matrices, as shown in Table 2.

**Table 2:** Judgment matrix.

Risk cost	O	D	P
O	1	1/7	1/2
D	7	1	5
P	2	1/5	1

The third step involves using the square root method (Eq. 31) to calculate the geo metric mean of the normalized elements in each row of the judgment matrix, denoted as ${w}_{i}$.

$${\text{w}}=\left[{w}_{1}, {w}_{2},{w}_{3}\right]=[0.1228, 0.9682, 0.2181]^{\prime}$$

31 $$ {\text{Feature}}\,{\text{matrix: C}} = \left[ {\begin{array}{*{20}c} {a_{11} } & {a_{12} { } \ldots } & {a_{1n} } \\ {a_{{\begin{array}{*{20}c} {21} \\ \vdots \\ \end{array} }} } & {{ }a_{{\begin{array}{*{20}c} {22} \\ \vdots \\ \end{array} }} { } \ddots { }} & \vdots \\ {a_{n1} } & {{ }a_{n2} { } \ldots { }} & {a_{nn} } \\ \end{array} } \right],w_{i}^{0} = \frac{{\left( {\mathop \prod \nolimits_{j = 1}^{n} a_{ij} } \right)^{\frac{1}{n}} }}{{\mathop \sum \nolimits_{i = 1}^{n} \left( {\mathop \prod \nolimits_{j = 1}^{n} a_{ij} } \right)^{\frac{1}{n}} }},{\text{ n}} = {1},{ 2} \ldots {\text{n}} $$

where ${a}_{ij}$ is the result of comparison between elements $i$ and $j$, and the relative weight ${w}^{0}$ of each risk cost can be obtained by normalizing ${w}^{0}$:

$${w}^{0}={[w}_{1}^{0}{,w}_{2}^{0},{w}_{3}^{0}]^\prime=[0.0938, 0.7396, 0.1666]^{\prime}$$

Finally, check the consistency of the judgment matrix. The consistency indicators are $C.I.$, $R.I.$ and $C.R.$ ($C.I.$ is the consistency index, $R.I.$ is the random index, and $C.R.$ is the consistency ratio), and the calculation of $C.I.$ is as follows:

32 $$C.I.=\frac{{\lambda }_{max}-n}{n-1}$$

where ${\uplambda }_{{\text{max}}}$ is the maximum eigenvalue of the judgment matrix, $n$ is the order of the judgment matrix, and the value of $R.I.$ refers to the table of average random consistency index shown in Table 3. The lookup table yields $n$=3, $R.I.$=0.52. $C.R.$ is calculated as follows:

**Table 3:** Average stochastic consistency indicators.

$n$	1		2	3	4	5	6	7	8	9	10	11	12	13	14
*R*.*I*	0		0	0.52	0.89	1.12	1.26	1.36	1.41	1.46	1.49	1.52	1.54	1.56	1.58

33 $$C.R.=\frac{C.I}{R.I.}$$

$C.R.$ < 0.1 is considered to pass the consistency test; if the conditions are not met, the judgment matrix must be checked and its value adjusted. $CR.=\frac{C.I}{R.I.}=\frac{0.0071}{0.52}=0.0136$ pass the consistency test. The specific results are shown in Table 4, so ${\alpha }_{1}$=0.0938, ${\alpha }_{2}$=0.7396, ${\alpha }_{3}$=0.1666.

**Table 4:** Results of the weight solution.

Risk cost	O	P	D	${{\varvec{w}}}_{{\varvec{i}}}$	${{\varvec{w}}}_{{\varvec{i}}}^{0}$
O	1	1/9	1/9	0.1228	0.0938
P	9	1	1	0.9682,	0.7396
D	9	1	1	0.2181	0.1666

$$C.I=0.0071 R.I.=0.52$$ $$C.R=0.0136$$

### Smoothing of the risk path

**Figure 11:**
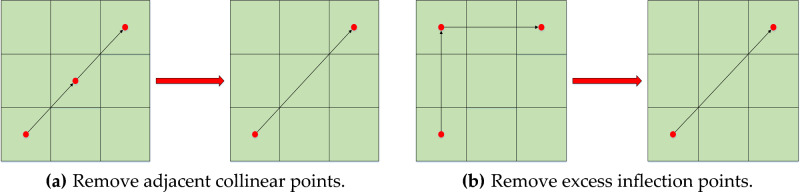
Schematic diagram of the Floyd algorithm. The Floyd algorithm is used for path smoothing to eliminate adjacent collinear points and redundant turning points.

The result of the A* algorithm is a string of path point coordinates. The generated path is the folding line "Z" route, which does not conform to the real application. The Floyd algorithm can optimize the path, remove the extra points and inflection points of the A* algorithm path, and combine collinear node^38^.This paper realizes the smooth risk path by improving the Floyd algorithm. The path-smoothing principle of the Floyd algorithm is shown in Fig. 11.

**Figure 12:**
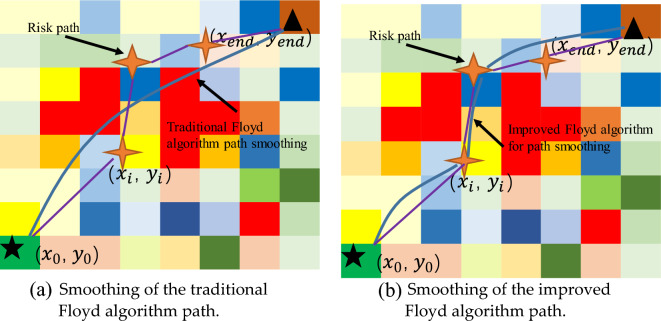
Smooth comparison of UAV paths in the risk environment. (**a**) Indicates that the traditional Floyd algorithm may smooth out the risk inflection points, causing the UAV's flight path to pass through excessively high-risk areas. (**b**) Indicates that the improved Floyd algorithm ensures flight safety while smoothing the path.

However, in a risky environment, the traditional Floyd algorithm has the possibility of removing low-risk path coordinates. The path coordinates determined by the Min-cost A* algorithm in the risk environment is to avoid the risk area, the traditional Floyd algorithm may smooth out the coordinates of some risk areas that need to be avoided, increasing the risk of drone flight, as shown in Fig. 12a. To solve this problem, this paper sets a risk threshold for the three-dimensional risk environment, and when the risk value exceeds this threshold, the original path point is retained, smaller than the risk threshold for smooth treatment, as shown in Fig. 12b.

## Simulation results

In this paper, our risk assessment model and a risk-based path planning algorithm are simulated in the urban risk map. First, the risk assessment model is applied to the actual environment to generate an airspace map. Based on the flight risk analysis, a low-risk flight path is generated and smoothed. The effectiveness of the algorithm is proved by comparing the algorithms and analyzing the experimental results.

### Establishing the experimental environment

**Figure 13:**
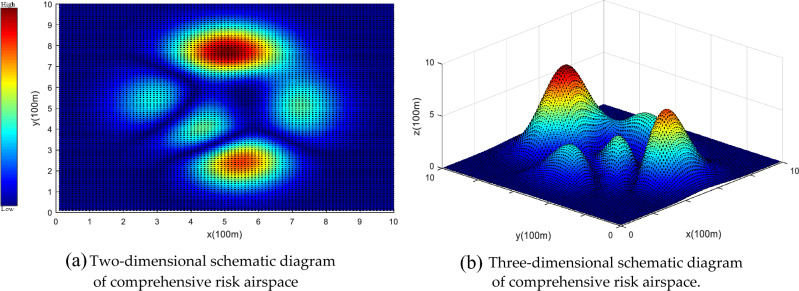
Comprehensive airspace risk simulation map. The brighter the color on the map, the higher the risk for the area.

The current study utilizes a multirotor unmanned aerial vehicle (UAV), specifically the DJI Phantom 4 Pro. Its main parameters are as follows: weight of 1.38 kg, maximum flight time of 30 min, maximum flight speed of 72 km/h, maximum flight altitude of 6000 m, minimum step length of 0.1 m, turning angle and climbing angle of ± 30°, and a turning radius of 10–15 m (in P mode, which refers to the normal flight mode). The main simulation environment for this article is as follows: 11th Gen Intel(R) Core(TM) i7-11800H @ 2.30 GHz, Windows 11 operating system, is simulated in MATLAB and Pycharm Community Edition. We selected a 1 km × 1 km urban area in Xi'an, China for airspace modeling, with each three-dimensional gas block element measuring 100 m × 100 m × 30 m. The selected area includes high-rise office buildings, residential buildings, and public facilities, representing the characteristics of a modern city. The area consists of a total of 652 buildings, with an average population density of 11,378 people per square kilometer^39^. Equations (1), (2), and (14) are used for risk assessment to generate a comprehensive risk simulation map that includes obstacle risk, death risk, and property loss risk, as shown in Fig. 13. Different color distributions on the map represent different risk distributions in the city. The brighter the color on the map, the higher the risk of the area. These high-risk areas mean a large population and more building distribution. The city risk can be estimated through the third-party risk assessment model and the city risk map can be generated, which is the basis for UAV path planning based on risk cost. In the following, we further perform a flight risk analysis, generate UAV paths based on urban risk maps, and verify the algorithm in this paper.

### Flight risk analysis

**Figure 14:**
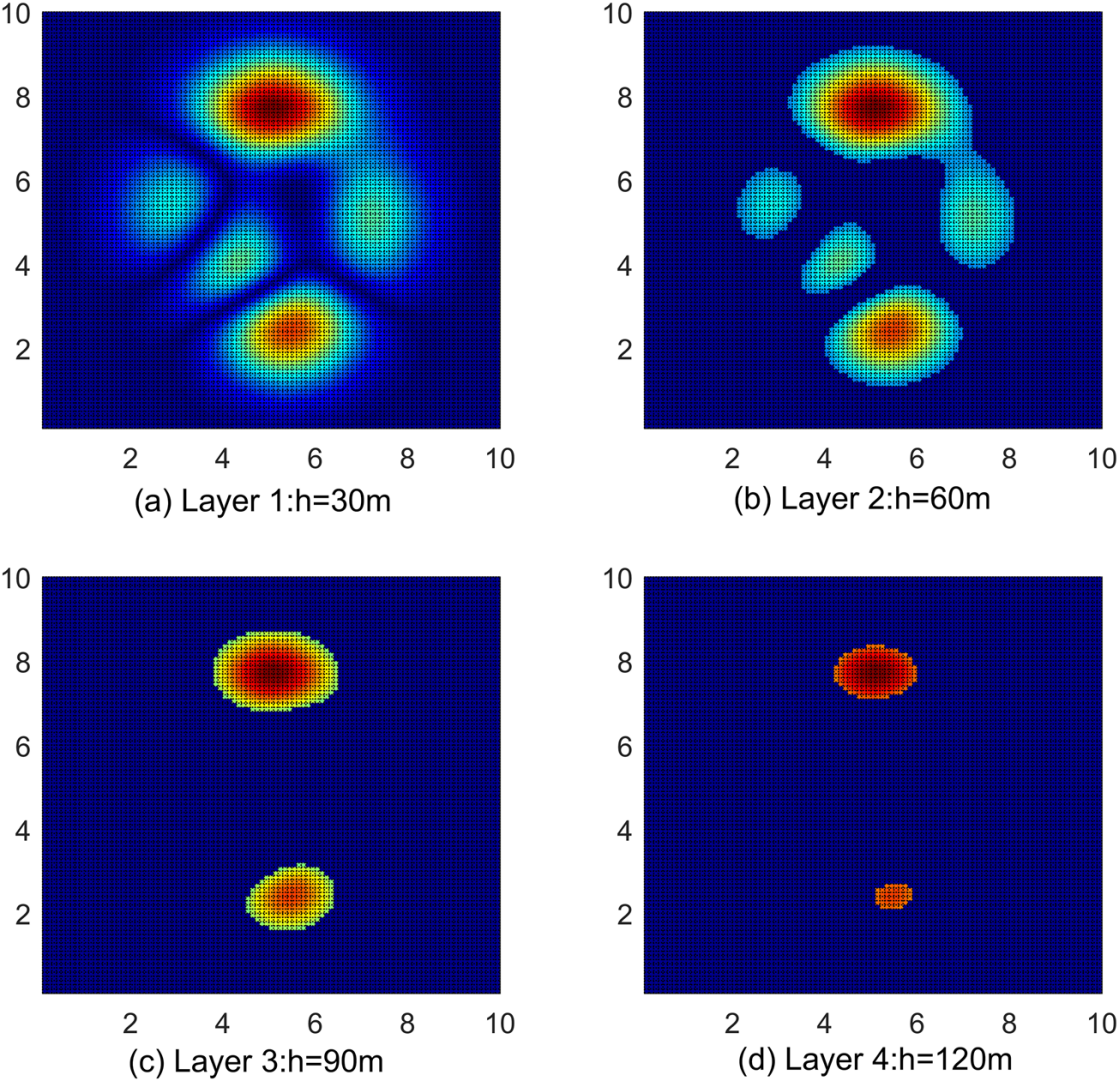
Risk cost map for different flight altitudes. The diagram illustrates risk zones at altitudes of 30 m, 60 m, 90 m, and 120 m. Due to factors such as buildings and population, lower-altitude maps display a higher density of risk areas.

In the UAV risk cost model, the weight factors of obstacle risk, death risk and property loss risk are 0.0938(${\alpha }_{1}$), 0.7396(${\alpha }_{2}$), and 0.1666(${\alpha }_{3}$), and the comprehensive risk cost of different flight layers is calculated. Within the restricted flight altitude of urban drones, the total flight cost decreases as the flight altitude increases, as shown in Fig. 14. In the airspace within 60 m, as shown in (a) and (b) in Fig. 14, the density of obstacles, population, and buildings is large. For example, the area near the map (5, 2), (6, 8) has the highest risk cost, then in the actual environment, such areas often include schools, office buildings, pedestrian streets, etc. When the flight altitude reaches more than 90 m, the total risk cost is significantly lower than within 60 m, as shown in (c) and (d) in Fig. 14. The reasons are as follows: the increased altitude of drones leads to a decrease in the risk of property damage, and the risk of obstacles and population density leads to a decrease in the risk of direct death, so the total cost of risk is reduced. However, the high-risk areas at flight altitudes of 90 and 120 m can still be clearly identified because there are still high-rise buildings at this altitude and the density of these buildings is related to the risk of death and accounts for 73.69%.

### Analysis of path planning methods

#### Effectiveness analysis of min-cost A* algorithm

**Figure 15:**
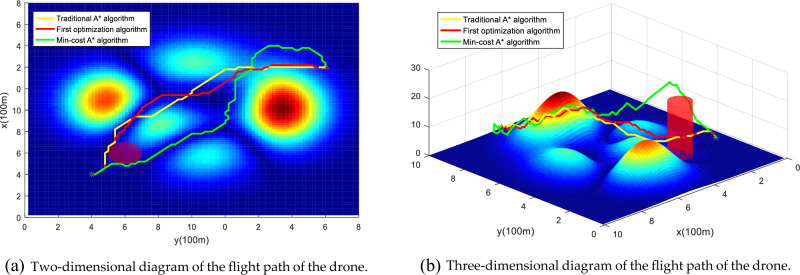
UAV flight paths with different algorithms. Note: Dark red in (**a**) indicates the “no-fly zone” and in (**b**) the cylinder indicates the three-dimensional space no-fly zone.

In 4.2.1, this paper integrates obstacle risk, death risk and property loss risk into the A* algorithm, and proposes the Min-cost A* algorithm through two improvements to obtain a low-risk flight path. In the simulation, a “no-fly zone” is added to further verify the effectiveness of the algorithm. The starting point is (20, 20, 5) and the endpoint is (90, 90, 7), and the experimental results show that the improved algorithm compares with the original A* and the risk of the path is significantly reduced, and it can effectively avoid high-risk areas in the city, as shown in Fig. 15. Yellow indicates the flight path of the traditional A* algorithm and red indicates the flight path of the first optimization algorithm. The first optimization algorithm mainly integrates the third-party risk cost into the cost function and heuristic function of the traditional A* algorithm to generate a low-risk flight path (Eqs. 25, 26). Green indicates the flight path of the Min-cost A* algorithm (Eq. 30). It can be seen in the figure that the improved algorithm can effectively avoid "no-fly zones" and high-risk areas near (4, 10), greatly improving flight safety.

**Figure 16:**
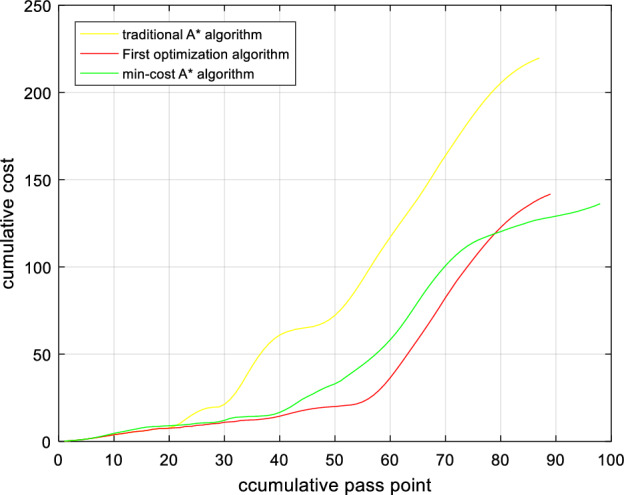
Path risk comparison. Once the number of search nodes exceeds 80, the Min-cost A* algorithm offers the lowest path risk.

**Figure 17:**
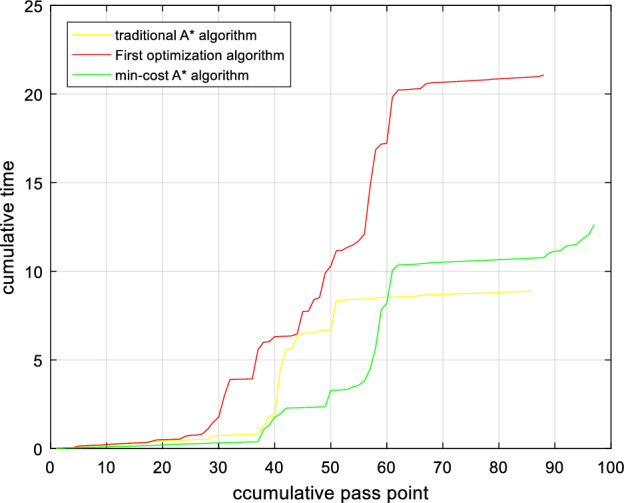
Algorithm time comparison. The Min-cost A* algorithm strikes a balance between path risk and search time.

The Min-cost A* algorithm proposed in this paper (in Eq. 28), based on the first optimization algorithm, a distance function based on the Euclidean distance and the Manhattan distance is added to improve the search speed of the algorithm) has a much lower cumulative risk than the traditional A* algorithm, as shown in Figs. 16 and 17. The experimental results show that compared to the traditional A* algorithm, the algorithm after the first optimization reduces the path risk by 40.47%, the algorithm search time is increased by 63.6%, the path risk of the Min-cost A* algorithm is reduced by 44.44%, and the time is only increased by 27.2% compared with the original A* algorithm. Therefore, the Min-cost A* algorithm proposed in this paper can ensure the effective implementation of the algorithm while reducing the risk of a UAV flight path.

**Figure 18:**
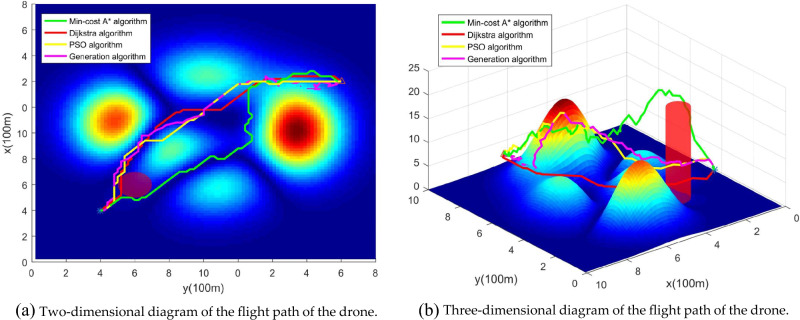
Comparison of Min-cost A* with other advanced algorithms. Note that this paper com pares Dijkstra algorithm, particle swarm optimization (PSO) algorithm, and genetic algorithm. In (**a**), deep red indicates “no-fly zones,” and in (**b**), cylindrical shapes represent three-dimensional airspace restrictions.

To comprehensively assess the relative performance of our algorithm in this paper, we add a comparison between the Min-cost A*algorithm and the Dijkstra algorithm, particle swarm optimization algorithm (PSO), and genetic algorithm, as shown in Fig. 18. Near the map coordinates (6, 6), the Dijkstra algorithm plans paths that cross the “no-fly zone,” and as we approach the endpoint, the genetic algorithm plans paths that cross some high-risk areas. Although the paths generated by the particle swarm optimization algorithm do not exhibit instances of crossing no-fly zones or high-risk areas, they do not make reasonable avoidance maneuvers for lower-risk areas near map coordinates (8, 8). In real-world scenarios, flight safety concerns in these areas still warrant attention. The path risks for different algorithms are presented in Table 5, and when compared to the Dijkstra algorithm, the particle swarm optimization algorithm, and the genetic algorithm, the Min-cost A* algorithm reduces path risks by 42.15%, 29.30%, and 45.31%, respectively. Additionally, it also produces the shortest paths, achieving a balance between flight path risk and path length.

**Table 5:** Comparison of Path Risks between Min-cost A* Algorithm and Other Algorithms.

	Min-costA* algorithm	Dijkstra algorithm	PSO algorithm	Genetic algorithm
Cumulative risk	140	242	198	256
Path length	1323.906	1450.361	1423.582	1336.054
Comparison with other algorithms: Risk reduction by Min-cost A*	0%	42.15%	29.30%	45.31%

Min-cost A* Algorithm shows a 0% decrease in risk when compared to itself.

#### Risk path smoothing

**Figure 19:**
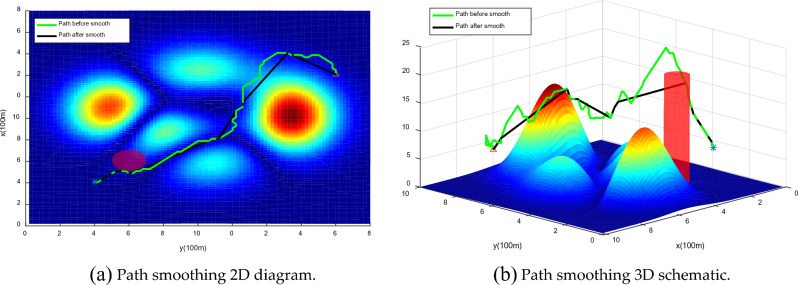
Path smoothing comparison chart. The smoothed path, while ensuring path safety, removes adjacent collinear points and unnecessary turning points.

In this paper, the UAV path is smoothed according to the smoothing strategy proposed in 4.3, and the simulation results are shown in Fig. 19. The experimental results show that under the premise of satisfying flight safety, the inflection point of the path is less than that before smoothing and the path is smoother.

#### Algorithm effectiveness verification

**Figure 20:**
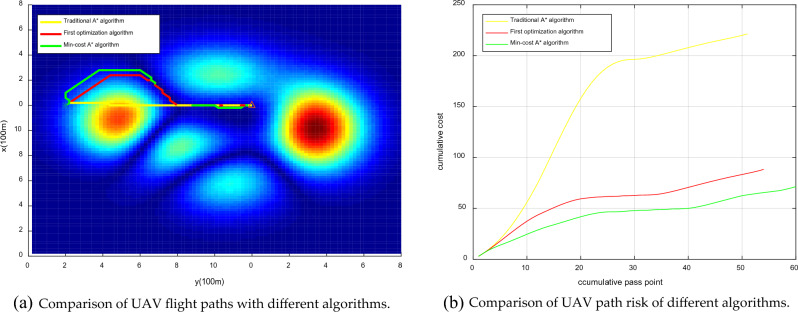
Comparison of the UAV flight path and path risk with different algorithms. This set of simulations starts at (60, 10, 5) and ends at (60, 60, 10).

**Figure 21:**
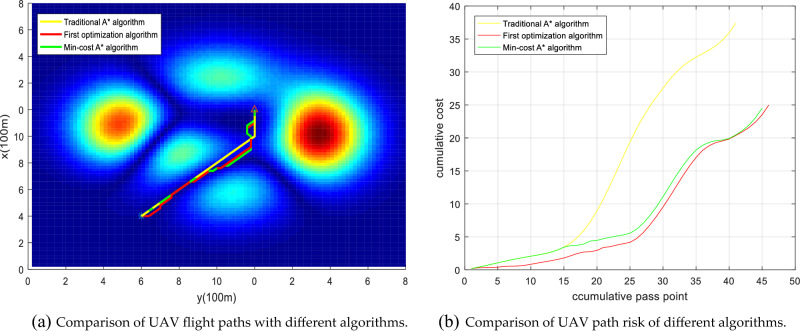
Comparison of the UAV flight path and path risk with different algorithms. This set of simulations starts at (20, 30, 8) and ends at (60, 60, 12).

**Figure 22:**
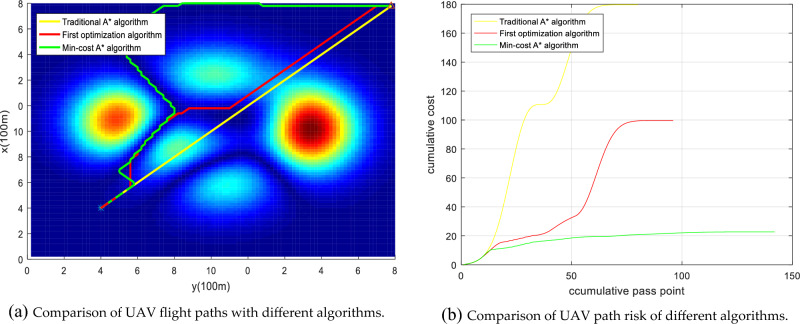
Comparison of the UAV flight path and path risk with different algorithms. This set of simulations starts at (20, 20, 25) and ends at (99, 99, 20).

**Figure 23:**
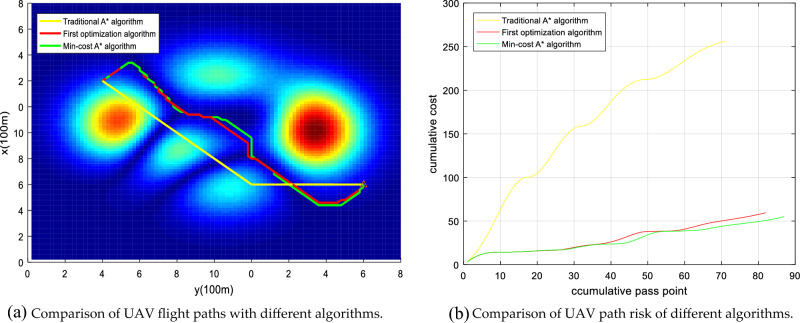
Comparison of the UAV flight path and path risk with different algorithms. This set of simulations starts at (70, 20, 6) and ends at (30, 90, 10).

Urban drones operate flexibly, so we set any flight start and end point for multiset experimental comparison, as shown in Figs. 20, 21, 22, 23. The experimental results show that compared to the traditional A* algorithm and the first optimization algorithm, the Min-cost A* algorithm can plan the flight path with the smallest risk, and the cumulative flight risk is much lower than that of the traditional A* algorithm, as shown in Table 6.

**Table 6:** Comparison of the experimental results of different path planning algorithms.

Calculation result	Compared to the traditional A * algorithm (risk reduction)				
Cumulative risk	Traditional A* algorithm	First optimization algorithm	Min-cost A* algorithm	First optimization algorithm (%)	Min-cost A* algorithm (%)
	225	80	60	64.44	73.33
	36	20	19	44.44	47.22
	180	100	23	222.22	436.11
	255	50	45	80.39	82.35

**Figure 24:**
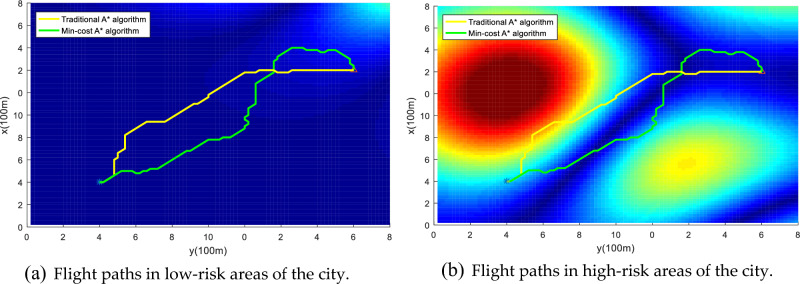
Different risk flight areas in the city. (**a**) Represents low-risk flying areas in urban areas, such as green spaces, and (**b**) represents high-risk flying areas in urban areas, such as pedestrian streets and residential areas.

**Figure 25:**
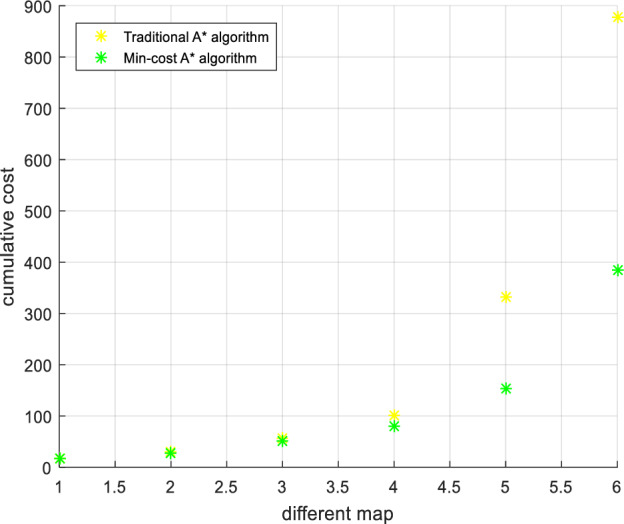
Comparison of total path risk. The Min-cost A* algorithm has lower path risk compared to the traditional A* algorithm, and this difference increases with the expansion of the map scope.

**Figure 26:**
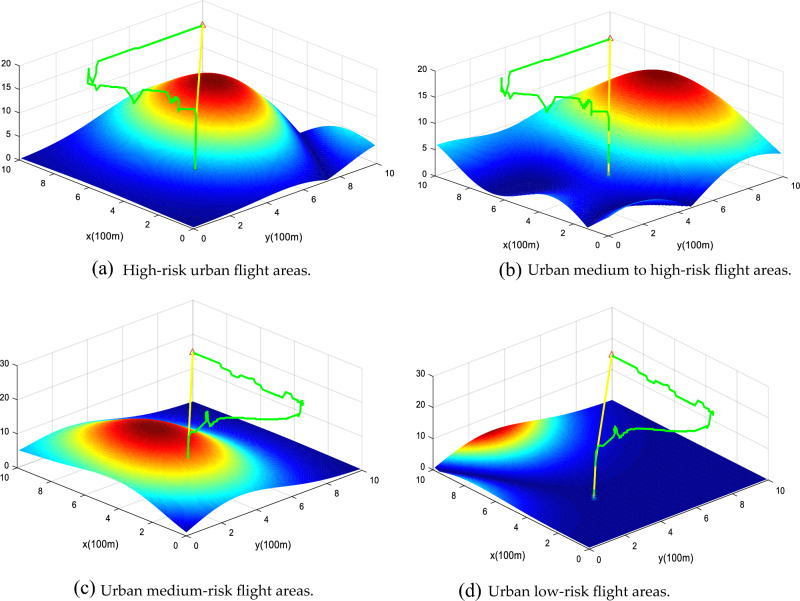
Schematic diagram of UAV path planning in different risk environments. Note: The yellow line indicates the flight path planned by the traditional A* algorithm, and the green line indicates the flight path planned by the Min-cost A* algorithm.

**Figure 27:**
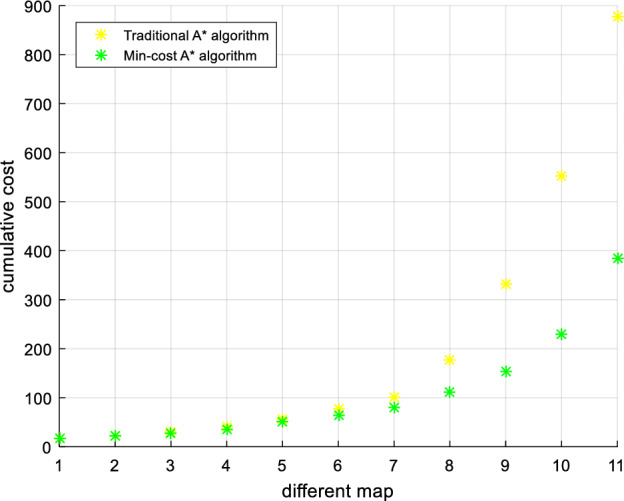
Path-risk comparison of different algorithms. The Min-cost A* algorithm has lower path risk compared to the traditional A* algorithm in any area of the city (high-risk, medium to high-risk, medium-risk, and low-risk flying areas).

In order to further verify the safety of the algorithm path in this paper, we compare the flight path of the traditional A* algorithm and the Min-Cost traditional A* algorithm in different risk areas, and the example map is shown in Fig. 24. In areas with low urban risk Fig. 24a (such as green areas), the total risk gap between the A* algorithm and the Min-cost A* algorithm is small, because such areas themselves have fewer buildings and population distribution, and the airspace is spacious. When the drone is operated in a high-risk area of city Fig. 24b (such as a pedestrian street), the total risk of the Min-cost A* algorithm path is significantly lower than that of the traditional A* algorithm, as shown in Fig. 25. Through multiple sets of simulations as shown in Fig. 26, the Min-cost A* algorithm can plan the lowest-risk flight path on any map, and the total risk of the Min-cost A* algorithm path is significantly lower than that of the traditional A* algorithm, as shown in Fig. 27 and Table 7.

### Optimal convergence analysis of the Min-cost A* algorithm

In Section “Effectiveness analysis of Min-cost A* algorithm”, we compare the Min-cost A* algorithm with the traditional A* algorithm and the first optimization algorithm. The experimental results show that the Min-cost A* algorithm reduces path risk by 44.44% compared to the traditional A* algorithm. After the second optimization, the search time of the Min-cost A* algorithm decreases by 36.4% from the original time. Additionally, we conduct comparative experiments between Min-cost A* and the Dijkstra algorithm, Particle Swarm Optimization (PSO), and Genetic Algorithm (GA). The results show that compared to the Dijkstra algorithm, PSO, and GA, the Min-cost A* algorithm reduces path risk by 42.15%, 29.30%, and 45.31%, respectively.

**Table 7:** Comparison of cumulative risk between the traditional A* algorithm and Min-cost A* algorithm on different maps.

City risk	High-risk		Medium to high risk		Medium-risk		Low-risk	
Cumulative risk	Traditional A* algorithm	Min-cost A* algorithm	Traditional A* algorithm	Min-cost A* algorithm	Traditional A* algorithm	Min-cost A* algorithm	Traditional A* algorithm	Min-cost A* algorithm
	890	395	550	220	320	150	180	100

In Section “Algorithm effectiveness verification”, we conduct comparisons by setting arbitrary starting and ending points and varying the map's risk levels. The experimental results indicate that regardless of changing the flight's starting and ending points, the Min-cost A* algorithm consistently plans low-risk flight paths. In maps with different risk levels, the Min-cost A* algorithm generates safer flight paths compared to the traditional A* algorithm, eliminating the algorithm’s randomness.

In conclusion, the Min-cost A* algorithm converges and is optimal because it plans the lowest-risk flight path, striking a balance between path risk evaluation and search time.

## Summary and outlook

### Summary

Drones are an extension of urban infrastructure and public services, and reducing the risk of drone operations is a key factor to attract investment in the opening of airspace. Aiming at the UAV path planning problem in urban flight environment, we establish a third-party risk model to evaluate the risk of obstacles, death, and property damage in the urban flight environment. A UAV path planning model based on third-party risk is established, and the effectiveness of the proposed algorithm is demonstrated through experimental simulation. The main conclusions are as follows:

By establishing a third-party risk model, the risk of obstacles, death, and property damage in the operation of UAVs in the urban environment can be evaluated, and the risk analysis of different flight altitudes can be carried out to determine the high-risk areas of the city.

The established third-party risk is added to the UAV path planning model, which can capture the risk factors in the urban environment, avoid flying in high-risk areas, and reduce flight path risk.

The proposed Min-cost A* algorithm can plan the minimum-risk flight path, and the experimental results show that the Min-cost A* algorithm reduces the path risk by 44.44% compared to the traditional A* algorithm. Through the improved Floyd algorithm, the smoothing of the urban risk path can be achieved under the premise of the minimum risk of the path.

The proposed path planning algorithm based on third-party risk provides a new heuristic function determination scheme for the cost-based path search method, which can improve the reliability of the flight path of the UAV and not fall into local optimum due to too large heuristic function estimates.

### Outlook

The Min-cost A* algorithm proposed in this paper significantly reduces the risk associated with urban drone flight paths. However, we will continue to conduct in-depth research into the following scenarios:

1. When there are a large number of dynamic obstacles in the environment, the Min-cost A* algorithm fails to consider the changing nature of these obstacles, making it unable to perceive and avoid them in real-time, thus increasing the risk of collisions. To address this issue, we will utilize the artificial potential field method to assign potential energies to targets and obstacles. This will transform the path planning problem into a gradient search problem in the potential field, allowing for better avoidance of dynamic obstacles.
2. In the case of large-scale maps and complex drone motion patterns, the Min-cost A* algorithm may face higher computational complexity. To address this issue, we will use a quadtree data structure to accelerate the search process. We’ll divide the search space into quadtree nodes and store information about the region represented by each node, reducing the number of nodes that need to be searched and thus reducing the computational complexity of the algorithm.
3. To address the problem of getting trapped in local minima in the aforementioned scenario, we will introduce the D* Lite algorithm for local path correction. This approach avoids unnecessary global research, improves computational efficiency, and enables quick adaptation to environmental changes.

### Supplementary Information

Supplementary Information.

## Data Availability

All data generated or analysed during this study are included in this published article.
