# Supplementary material for: UAV path planning based on third-party risk modeling
Source: Sci Rep. 2023 Dec 14;13:22259. doi: 10.1038/s41598-023-49396-4 (PMC10721927; doi:10.1038/s41598-023-49396-4)
Supplement: Supplementary file 1 — Supplementary Information. [file 41598_2023_49396_MOESM1_ESM.pdf]

Terms	Definitions and descriptions	Terms	Definitions and descriptions
O	Obstacle risk	P	Property Loss Risk
$V_{obstacle}(r)$	The volume of obstacles in a three-dimensional gas block cell with side length r	$V_{surround}(r)$	The volume of a three-dimensional gas block element with side length r.
D	Death risk	ξ	The skin penetration degree
D_1	Direct death risk	r_{UAV}	The radius of the drone
D_2	Indirect death risk	r_{Human}	The radius of the person
P_{crash}	The probability that the drone fails to fall	P_{impact}	The probability that the drone falls to hit people
N_{hit}^{p}	The number of people hit by the drone	P_D^p	The mortality rate in the drone accident
E_{imp}	The impact kinetic energy	s_c	The occlusion factor
σ_p	The density of the population	σ_b	The density of the building
N_D^p	The average number of casualties caused by a drone colliding with a building.	A_P	The contact area (A_P) of the crash between the falling drone and the person
N_{b_impact}	The number of buildings impacted by the drone's fall	S_{hit}	The area expected to be hit by the drone
S_{b_hit}	The estimated drone impact area	m	The mass of the drone
Δh	The change of the drone flight altitude	$\alpha_{1、2、3}$	The weight factor of Obstacle risk, death risk and Property risk
φ_{max}	The maximum steering angle of the drone	θ_{max}	The maximum climb angle of the drone
r_{min}	The limit turning radius	v_{min}	The minimum flight speed
n_{ymax}	Indicates the maximum normal overload of the drone	t_{max}	The maximum UAV endurance time
H_{min}	The minimum flight altitude	H_{max}	The maximum flight altitude
l	The minimum step length	g	Gravity acceleration
$f(n)$	The comprehensive priority of node n,	$g(n)$	The cost of the node n from the starting point
$h(n)$	The estimated cost of node n from the endpoint	$G_{cost}(n)$	The cost function of the Min-cost A* algorithm
$H_{cost}(n)$	The heuristic function of the Min-cost A* algorithm	$H_{cost}^{*}(n)$	The optimized heuristic function
$H_{cost}^{*}(n)$	The optimized heuristic function	$d(n)$	The Euclidean distance
A	The Manhattan distance from the current node to the endpoint	B	The Manhattan distance from the current node to the starting point
w	Judgment matrix	C	Feature matrix
$C.I.$	The consistency index	$R.I.$	The random index
$C.R.$	The consistency ratio	w^0	The relative weight
$\lambda_{\max}$	The maximum eigenvalue of the judgment matrix	a_{ij}	The result of comparison between elements i and j
